# Intraductal Radiofrequency Ablation Followed by Locoregional Tumor Treatments for Treating Occluded Biliary Stents in Non-Resectable Malignant Biliary Obstruction: A Single-Institution Experience

**DOI:** 10.1371/journal.pone.0134857

**Published:** 2015-08-05

**Authors:** Xu-Hua Duan, Yan-Li Wang, Xin-Wei Han, Jian-Zhuang Ren, Teng-Fei Li, Jian-Hao Zhang, Kai Zhang, Peng-Fei Chen

**Affiliations:** Department of Interventional Radiology, The First Affiliated Hospital, Zhengzhou University, No. 1, East Jian She Road, Zhengzhou, 450052, Henan Province, People’s Republic of China; Texas A&M Health Science Center, UNITED STATES

## Abstract

**Objectives:**

To determine the safety and feasibility of intraductal radiofrequency ablation (RFA) followed by locoregional tumor treatments in patients with non-resectable malignant biliary obstruction and stent re-occlusion.

**Methods:**

Fourteen patients with malignant biliary obstruction and blocked metal stents were studied retrospectively. All had intraductal RFA followed by locoregional tumor treatments and were monitored clinically and radiologically. The practicality, safety, postoperative complications, jaundice remission, stent patency and survival time were analyzed.

**Results:**

Combination treatment was successful for all patients. There were no severe complications during RFA or local treatments. All patients had stent patency restored, with a decline in serum bilirubin. Three patients had recurrent jaundice by 195, 237 and 357 days; two patients underwent repeat intraductal RFA; and one required an internal-external biliary drain. The average stent patency time was 234 days (range 187-544 days). With a median follow-up of 384 days (range 187-544 days), six patients were alive, while eight had died. There was no mortality at 30 days. The 3, 6, 12 and 18 month survival rates were 100%, 100%, 64.3% and 42.9%, respectively.

**Conclusion:**

Intraductal RFA followed by locoregional tumor treatments for occluded metal stents is safe and practically feasible and potential increase stent patency and survival times.

## Introduction

In patients suffering with unresectable malignant biliary obstruction with a life expectancy longer than 3 months, stent placement by endoscopic retrograde cholangiopancreatography (ERCP) or percutaneous transhepatic cholangiodrainage (PTCD) is the standard technique to ensure continued biliary drainage [1, 2]. However, tumor ingrowth, epithelial cell hyperplasia, clot accumulation and biliary sludge, amongst other complications, lead to a 50% stent re-stenosis rate within 6 months of stent insertion [2]. Traditionally, insertion of internal-external drains, balloon dilation, and stent replacement have been used to treat stent obstruction and re-stenosis [3, 4]. With the technological development of radiofrequency ablation (RFA), intraductal RFA catheters have played an increasingly important role in treating biliary stent occlusion [2, 5]. Previous clinical studies have demonstrated the safety and efficacy of this novel RFA catheter, which can be conducted endoscopically or by percutaneous transhepatic cholangiography (PTC) [2, 5].

Intraductal RFA is a newly emerging technique that delivers heat energy directly to neoplastic tissues to achieve tumor necrosis and to prolong biliary patency [2, 5, 6]. However, the heat penetration depth in intraductal RFA is limited and cannot achieve radical tumor elimination. In patients with malignant biliary obstruction, the median patency time of a metallic biliary stent is 6–9 months [7]. Unfortunately, patients experience complications from recurrent biliary obstruction due to local tumor progression, despite intraductal RFA and drainage. There are a few reported cases of the application of percutaneous intraductal RFA for clearance of occluded stents [8, 9], but tumor control after intraductal RFA in malignant biliary obstruction was not reported. Transcatheter arterial chemoembolization (TACE) following PTCD and stent implantation may significantly contribute to the survival time of patients with malignant biliary obstruction, and may improve stent patency [10, 11].

We undertook a retrospective analysis of data from 14 patients to investigate the safety and feasibility of intraductal RFA in biliary stent occlusion in patients with malignant biliary obstruction. We extended our analysis to explore if locoregional tumor treatments including TACE and intra-arterial chemotherapy with embolization of tumor-feeding arteries following intraductal RFA could increase the survival time for these patients.

## Materials and Methods

### Ethics statement

The study was approved by the ethics committee of Biomedical Research of the First Affiliated Hospital of Zhengzhou University and written informed consent was obtained from all the patients. The procedures followed were in accordance with the Helsinki Declaration of 1975, as revised in 1983.

### Subject selection

From January 2012 to November 2014, the records of patients with malignant biliary obstruction who had undergone PTCD and inner stent placement in our hospital were retrospectively analyzed. Fourteen patients with stent blockages who completely met the following eligibility criteria were recruited to this study: (i) forceps biopsy of the bile duct, or imaging examination to confirm the diagnosis of malignant biliary obstruction; (ii) previous PTCD and inner non-covered stent (Micro-Tech, Nanjing, China) placement; (iii) no indications for surgery or refusal to undergo surgery; (iv) no previous RF ablation of the malignant biliary strictures. The exclusion criteria were as follows: (i) tumor load of >70%; (ii) partial or complete main portal vein tumor thrombus; (iii) a Child-Pugh class C status, serum creatinine >2× baseline, or ECOG performance status of >1 at one month after intraductal RFA; (iv) intractable severe blood coagulation dysfunction; (v) massive refractory ascites; (vi) refusal to take part in the study.

Fourteen patients (ten men and four women) with a median age of 59 years (range 50–73 years) were included. All patients underwent imaging with CT or MRI to evaluate the cause of biliary obstruction before stent insertion. The underlying diseases comprised seven cases of biliary tract cancer (three with liver metastases), four cases of hepatic carcinoma, and three cases of gallbladder adenocarcinoma with liver metastases. All locoregional tumor treatments and intraductal RFA were performed by the same team of doctors.

### PTC biopsy and biliary stent implantation

**Fig 1:**
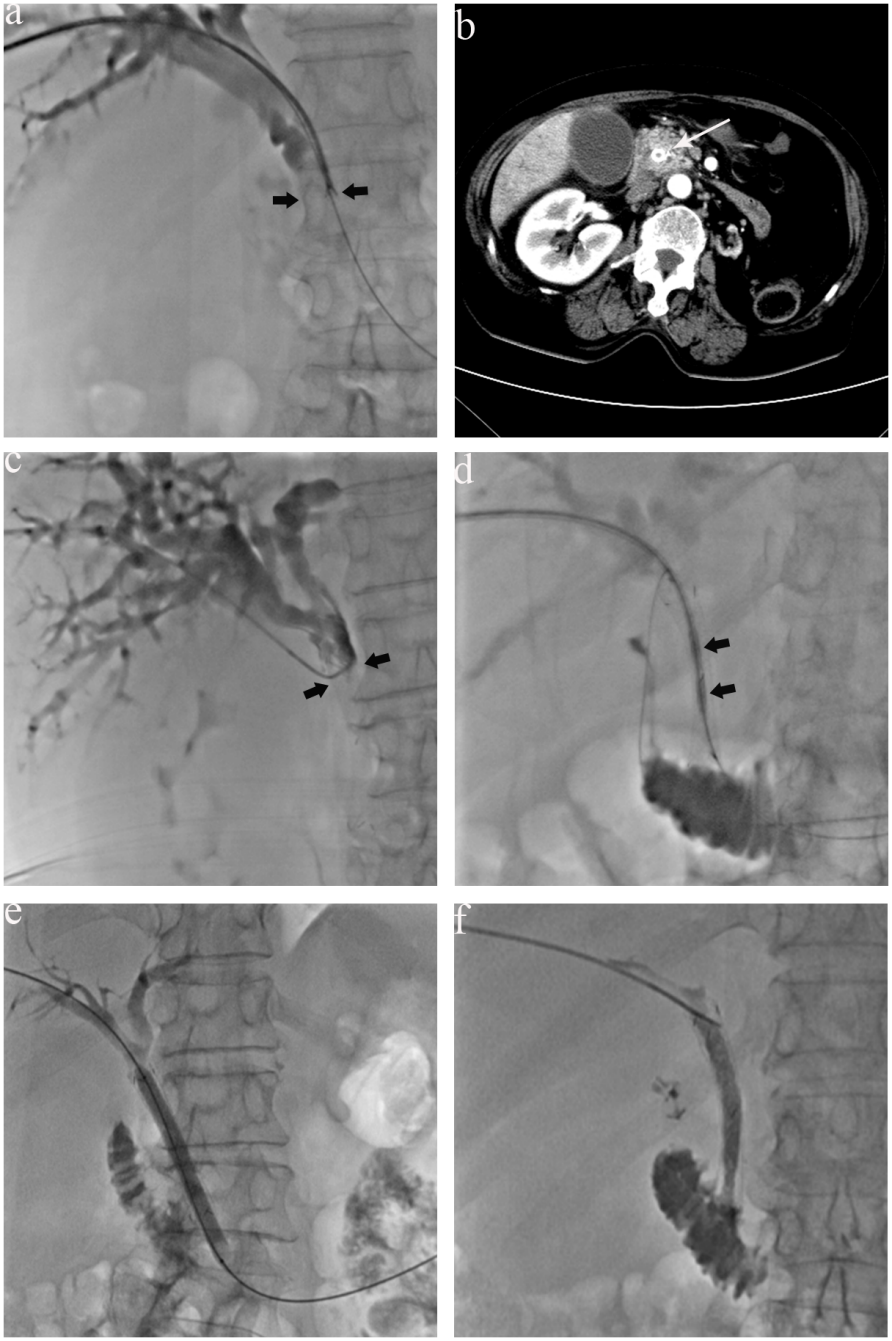
A 73-year-old woman with a highly differentiated adenocarcinoma of the middle third of the bile duct was underwent PTCB for pathogic diagnosis, at the site of biliary obstruction due to the tumor (arrows) (a). (b). Four months after biliary stent implantation, a contrast-enhanced CT showed dilated intrahepatic biliary ducts, a narrowing of the diameter of stent at the middle bile duct, and enlargement of the size of the tumor with obvious enhancement (long arrow). (c) Preablation PTC revealed stent blockages (arrows). (d) Intraductal RFA was performed with a percutaneous RFA catheter using a 0.035 inch guide wire. The two electrodes (arrows) of the RFA catheter were positioned in the area of the occlusion. (e) A balloon catheter moved back and forth through the stent into the duodenum to remove ablated tissue and debris from the stent. (f) Post-ablation PTC revealed the obstruction has been relieved.

All patients underwent PTC and percutaneous transhepatic cholangiography biopsy (PTCB) before stent implantation in the initial procedure. The procedure was performed by transhepatic puncture of a branch of the intrahepatic bile duct with a 21-gauge needle, under digital subtraction angiography (DSA) guidance (Artis Zeego, Siemens, Munich, Germany). PTC was then performed to visualize the location, extent and degree of bile duct obstruction using a percutaneous transhepatic cholangiography puncture set (Cook Inc., Bloomington, IN, USA). A 5 F Cobra catheter (Cook Inc.) was introduced into the bile duct with a 0.035 inch guide wire (Cook Inc.). The catheter and guide wire were passed through the stenosis simultaneously into the duodenum or jejunum. The guide wire was then replaced with a 0.035 inch stiff guide wire (Cook Inc.), and a 9 F catheter sheath was advanced along the stiff guide wire with 8 F biopsy forceps passed through the sheath. The sheath was pushed forward forcefully against the upper segment of the stenosis, while the biopsy forceps were introduced to the lesion through the introducing sheath. The PTCB was performed according to Li et al. [12] (Fig 1a). Following PTCB, an inner non-covered stent (Micro-Tech) was placed. Next, an 8.5 F internal–external biliary drainage tube (Cook Inc.) was implanted though the stent to the duodenum until the maximal decrease in serum bilirubin level was achieved.

### Intraductal RFA

After biliary stent implantation, patients were followed up by clinical examination, liver function tests and CT scans to rule out cholangitis or stent obstruction (Fig 1b). If stent occlusion occurred during the follow-up, intraductal RFA would be conducted. PTC was performed again to visualize the location, extent and degree of stent obstruction (Fig 1c). Using a 0.035 inch guide wire, the RFA catheter was placed such that the device’s working tip was positioned in the area of the occlusion (Fig 1d). Intraductal RFA was performed with a percutaneous RFA catheter (Habib EndoHPB, EMcision Limited, London, UK), which is connected to a compatible RF generator. Ten watts (W) of RF energy was applied for 2 min using a RF generator (1500X, RITA Medical Systems Inc., Fremont, California, USA), which has a frequency of 400 kHz. The ablation time was 1–2 min, and the location of the catheter was changed after a 1 min pause. Depending on the length of the stricture, sequential intraductal RFA were applied to treat the whole length of the stricture with an overlap of treated areas of approximately 1 cm. After intraductal RFA, an 8 mm × 4 cm balloon (Bard Peripheral Vascular, Inc, Tempe, AZ, USA) was moved back and forth through the stent into the duodenum to remove ablated tissue and debris from the metal stent as described by Pai et al. [8] (Fig 1e and 1f). Finally, external biliary decompression with an internal-external biliary drain was performed until the maximal decrease in serum bilirubin level was achieved.

After intraductal RFA, patients were given prophylactic antibiotics, liver-protective and symptomatic treatments. At 1 week and 1 month after the operation, remission of jaundice, short-term complications, and the serum bilirubin concentrations were recorded.

### Locoregional tumor treatments

**Fig 2:**
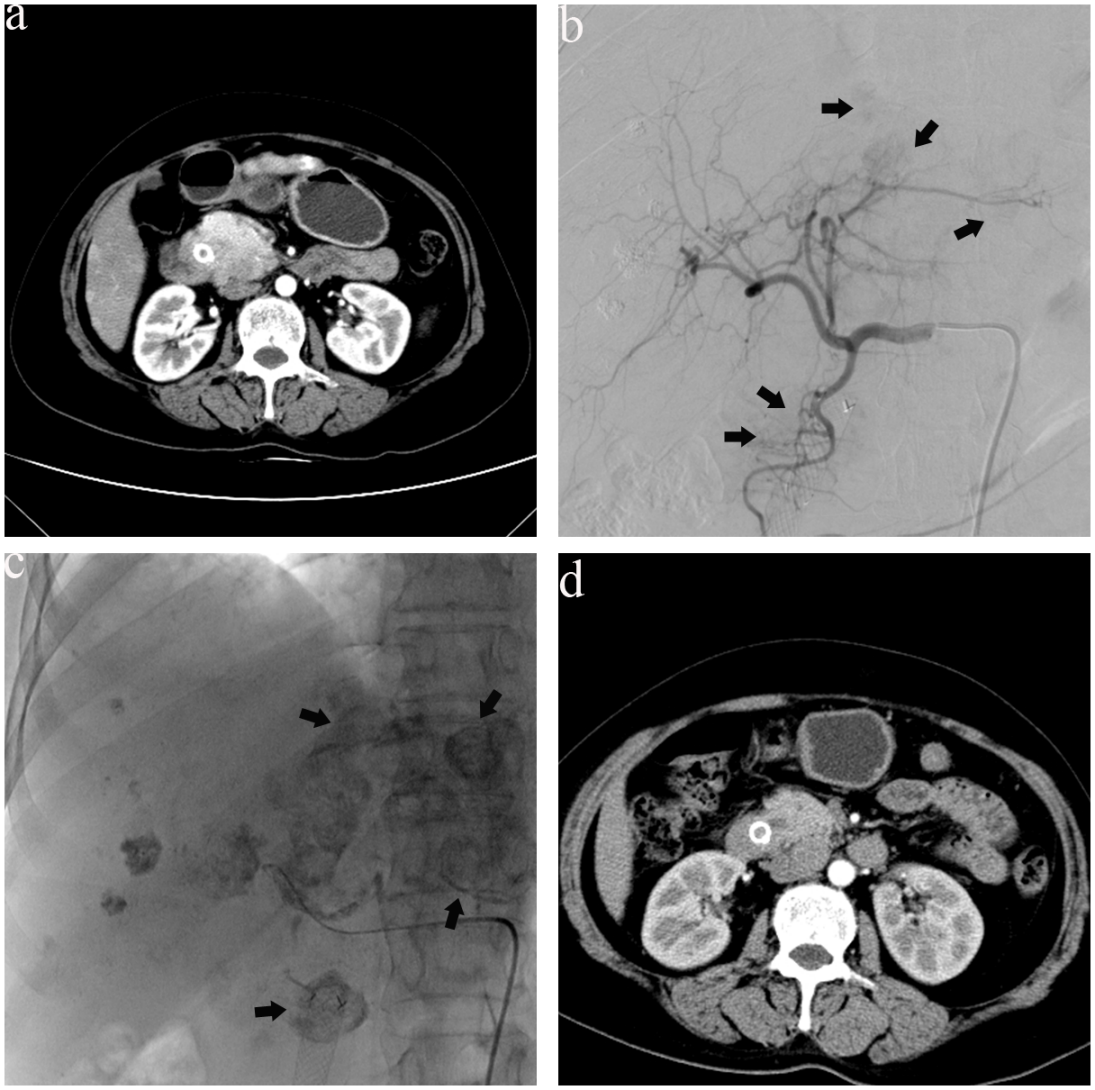
A 69-year-old man with a poorly differentiated adenocarcinoma of the middle third of the bile duct suffered stent blockage five months after stent implantation. (a) One month after intraductal RFA, CT showed the tumor was enlarged with obvious enhancement, plus liver metastases. (b) Hepatic artery angiography revealed multiple metastases nodules in the liver (arrows). (c) After the second TACE, lipiodol depositions were found in the tumor (arrows) and liver metastases. (d) CT images obtained at 3-month follow-up showed the tumor had reduced in size.

Contraindications to locoregional tumor treatments included Child-Pugh class C, a serum total bilirubin >53.5 μmol/L, serum creatinine >2 × baseline, and ECOG performance status >1. Locoregional tumor treatments were included TACE and intra-arterial chemotherapy with embolization of tumor-feeding arteries (Fig 2). Briefly, a 5.0 F RH catheter (Terumo, Tokyo, Japan) was used to perform a selective arteriogram of the celiac and superior mesenteric arteries to locate all tumor-feeding arteries. On the basis of the result of PTCB, we performed TACE with 30–50 mg pirarubicin mixed in 10–20 mL of lipiodol (Guerbet, Roissy, France) in patients with hepatocellular carcinoma (HCC); and intra-arterial chemotherapy with 0.8–1.2 g gemcitabine and 100–120 mg oxaliplatin and embolization of the tumor-feeding arteries with gelatin sponge particles (350–560 μm; Alicon, Shanghai, PR China) in patients with cholangiocarcinoma (CCA) as Gusani et al. [13] described. We implemented TACE with 10–15 mg mitomycin mixed in 5–10 mL of lipiodol in patients with gallbladder adenocarcinoma with liver metastases as described by Bode et al. [14]. In patients with liver metastases, we embolized the hepatic tumor-feeding arteries with 5–10 mL of lipiodol. Chemotherapeutic drug choice and medication dosage were made based on the patient’s previous treatment history, laboratory profile, tumor size and functional status.

### Efficacy evaluation and follow-up

After discharge, stent patency and patient survival were recorded using follow-up outpatient visits or telephone interviews. All 14 patients were followed up once a month after the operation. Patients underwent clinical examination, liver function tests and CT scans to rule out cholangitis or biliary obstruction. If stent re-occlusion occurred, another intraductal RFA followed by TACE was conducted.

Statistical analysis was performed using SPSS 13.0 (SPSS, Chicago, IL, USA). Data are presented as mean ± standard deviations of the mean, or median with range. The survival curves were constructed by the Kaplan-Meier method. The paired Student’s t-test was used to determine statistically significant differences mean bilirubin levels between pre- and post-procedure.

## Results

### Patient characteristics

The characteristics of the patients are shown in Table 1. PTCB followed by stent placement and intraductal RFA followed by locoregional tumor treatments were successfully performed in all 14 cases. The diagnosis at PTCB included three cases of CCA at the middle third bile duct (one with liver metastases), two cases of CCA at the lower third of the bile duct, one case of Bismuth I type CCA and one case of Bismuth IV (both with liver metastases), four cases of HCC, and three cases of gallbladder adenocarcinoma with liver metastases. The mean operating duration of intraductal RFA was 35 ± 14 min and the mean length of the biliary obstruction was 4.4 ± 0.6 cm. The average number of times locoregional tumor treatment was performed was 2.9 times (range 2–4).

**Table 1:** Overview of all patients undergoing intraductal RFA followed by locoregional tumor treatments for stent-occlusion and malignant biliary obstruction.

Patient	Sex/age	Tumor(staging)	No. of RFA	Length of the obstruction (cm)	No. of locoregional tumor treatments	Follow-up (days)	Outcome
1	M/73	Distal CCA*(T3N1M0)	2	5.2	3	523	Alive
2	F/50	Distal CCA(T2N1M0)	2	4.7	3	534	Alive
3	F/73	Distal CCA(T2N1M0)	1	3.8	3	511	Alive
4	M/65	Distal CCA(T2N1M0)	1	4.4	3	478	Dead
5	M/53	DistalCCA(T2N1M0)	1	4.9	2	484	Dead
6	M /46	CCA Bismuth I*(T3N0M0)	1	5.3	4	442	Alive
7	M /56	CCA Bismuth IV*(T3N1M0)	1	4.2	3	234	Alive
8	M /64	HCC(T3N1M0)	1	3.8	3	187	Dead
9	F/57	HCC(T3N1M0)	1	3.7	3	422	Dead
10	F/72	HCC(T3N0M0)	1	4	3	334	Dead
11	M/61	HCC(T3N0M0)	1	3.5	4	544	Alive
12	M /52	Gallbladder adenocarcinoma*(T3N1M0)	1	5.1	2	218	Dead
13	M /58	Gallbladder adenocarcinoma*(T3N0M0)	1	4.9	2	278	Dead
14	M /51	Gallbladder adenocarcinoma*(T3N0M0)	1	4.2	2	308	Dead

The number of follow-up months denotes the months from the first RFA in each patient.

Locoregional tumor treatments included TACE or superselective intra-arterial chemotherapy and embolization of tumor-feeding arteries.

*: These patients had liver metastases. F: Female; M: male; CCA: cholangiocarcinoma; HCC: hepatocellular carcinoma; RFA: intraductal radiofrequency ablation.

### Outcomes and follow-up

No patient suffered intraoperative discomfort or postoperative complications such as liver failure, pancreatitis, biliary hemorrhage, biliary fistula formation, biliary infection, hemothorax, pneumothorax, cholothorax, penetrating liver wounds or biliary peritonitis. After intraductal RFA, four patients presented with symptoms of cholangitis, including abdominal pain (n = 3), and chills and fever (n = 2); these resolved with antibiotics and conservative management. After locoregional tumor treatments, 12 patients presented with minor complications suggesting post-embolization syndrome, such as flank pain (n = 9), vomiting (n = 7), and fever (n = 5); these settled with the use of liver-protective drugs such as (compound glycyrrhizin injection) and conservative measures. One week after intraductal RFA, the mean serum bilirubin in all patients had decreased from 313 ± 103.4 μmol/L to 113 ± 59.1 μmol/L (P<0.001) and had further decreased to 39.5 ± 15.4 μmol/L (P<0.001) at 1 month after the procedure. In the follow-up CT images, cholangiectasis improved markedly after biliary tract reconstruction, with declines in serum total bilirubin and direct (conjugated) bilirubin. Three patients had recurrent jaundice by 195, 237 and 357 days; two underwent repeat intraductal RFA and one had an internal-external biliary drain sited, without further intraductal RFA. The average stent patency time was 234 days (range 187–544 days) in the 14 cases. The rate of stent-reblockage after intraductal RFA was 21.4% (3/14).

**Fig 3:**
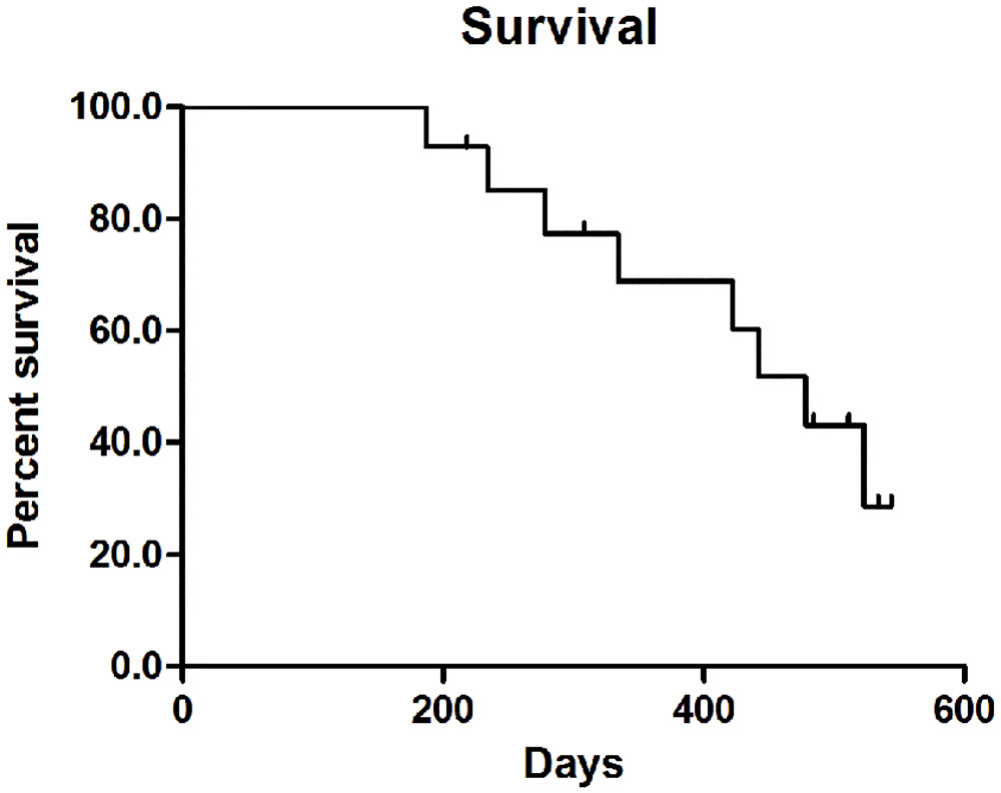
Survival curve for the intraductal RFA followed by locoregional tumor treatments as treatment for occluded biliary stents in malignant biliary obstruction.

Fig 3 shows the Kaplan-Meier curves depicting survival trends. Of the fourteen patients, six are alive and eight patients have died, at a median follow-up of 384 days (range 187–544 days). The 3, 6, 12, and 18 month survival rates were 100% (14/14), 100% (14/14), 64.3% (9/14) and 42.9% (6/14), respectively. One patient with CCA Bismuth IV with liver metastases died at 187 days, and one patient with HCC died 234 days after the procedure due to liver failure. The remaining six patients died of disease progression. Stent patency was preserved even in those patients who died.

## Discussion

The management of occluded primary metal biliary stents includes the use of different stent designs, endobiliary photodynamic therapy, mechanical cleaning of the stent with a balloon, insertion of internal-external drains through the blocked segment, and insertion of a second stent (whether covered stent, uncovered stent or plastic) through the primary occluded stent by balloon dilatation of the blocked segment [3, 4, 15]. Despite attempts to find a potential solution to problem of stent occlusion, little progress has been made in terms of improving the duration of stent patency [15].

RFA has been widely used in recent years as a first-line therapy for patients with small HCC who cannot undergo surgical resection or liver transplantation [16, 17]. It utilizes heat to achieve contact coagulative necrosis of the surrounding tissue. More recently, this technique has been recognized for its potential in the palliative treatment of malignant biliary obstruction [2]. Within the bile duct, RFA appears to lead to improved stent patency by decreasing tumor ingrowth and benign epithelial hyperplasia [2]. The introduction of the bipolar RFA catheter has opened up the possibility of a new type of treatment for bile duct carcinoma. Steel et al. [2] performed endoscopic intraductal RFA in 22 patients with malignant obstructive jaundice and reported curative effects, no severe postoperative complications, and low early and late stent-blockage rates, and concluded that this technique was safe and feasible for clinical use. Similar to endobiliary (via endoscopic route) RFA, the RFA catheter inserted over the guide wire via the percutaneous route can also be used as an adjunctive therapy in management of malignant biliary obstruction [18].

Mizandari et al. [5] successfully performed intraductal RFA via PTC followed by stenting under fluoroscopic guidance in 39 patients with malignant obstructive jaundice. Their results demonstrated the feasibility, safety and effectiveness of the PTC ablation technique. To date, there is no reported case that compared advantages and disadvantages of the application of percutaneous or endobiliary RFA for malignant biliary obstruction or clearance of occluded stents. Mizandari et al. [5] suggested that the percutaneous approach may be applied following biliary decompression with minimal discomfort to the patient [5]. Initial positive experiences with intraductal RFA for the clearance of occluded stents were reported by Pai et al. [8]. We believe it would be minimal discomfort to the patient and easy to intraductal RFA without concern endoscopy is not available as Pai et al. [8] reported. Due to the expensive cost of intraductal RFA catheters and the fact that intraductal RFA technology is not universally available, many clinicians have still focused on the use of expandable metal stents in patients with unresectable malignant biliary obstruction. The feasibility and clinical utility of intraductal RFA for the clearance of occluded stents is therefore an important clinical topic.

The safety, feasibility and efficacy of percutaneous intraductal RFA application for clearance of occluded metal stent in malignant biliary obstruction had been evaluated by Pai et al. [8]. This group successfully treated nine patients, of whom six were alive with a median follow-up of 122 days (range 50–488 days), with a median stent patency of 102.5 days (range 50–321 days). In the present study, 10 W of intraductal RF energy was applied for 2 min as described by Pai et al. [8]. All patients in our study had their stent patency restored successfully without the use of secondary stents, and with a median follow-up of 384 days (range 187–544 days); median stent patency was 234 days (range 187–544 days). The complications described in other studies [2, 5, 8], including extension of the RFA burn into adjacent structures, hemorrhage, bile duct perforation, bile leak and pancreatitis, were not observed. These results demonstrate that the parameters used in this study are safe, and add weight to previous reports that intraductal RFA via PTC for clearance of occluded metal stents in malignant biliary obstruction is safe and feasible.

Monga et al. [19] reported a case of common bile duct carcinoma treated with intraductal RFA. Two weeks after the procedure, choledochoscopy indicated that RFA was able to induce some destruction of local tumor tissue. Due to the limited heat penetration depth in intraductal RFA, it remains a palliative treatment for malignant biliary obstruction, especially for those patients who have tumors that involve the bile duct but do not originate from the bile duct epithelium (such as gallbladder carcinoma and HCC). Local coagulative necrosis caused by RFA only delays tumor growth within the stent, without controlling tumor beyond its confines. This may be the reason for the high rate of stent re-blockage after initial intraductal RFA—noted to be as high as 33.3% (3/9) in the study of Pai et al. [8], and 13.6% (3/22) in a study reported by Steel et al. [2]. Using CT and DSA imaging, we attribute re-blockage in our study to tumor ingrowth.

Successful biliary drainage as a palliative therapy is associated with improvement in quality of life in cases of malignant obstructive jaundice [20]. With intraductal RFA treatment of occluded biliary stents, and the associated alleviation of icterus and improvement of liver function reserves, patients are more likely to be able to undergo local tumor treatments such as chemotherapy or radiotherapy that may prolong their survival. Locoregional tumor treatments such as TACE are minimally invasive options that may, individually or in combination, achieve the best balance between successful tumor eradication and maximal preservation of liver function [21]. In a study by Qian et al. [11], 22 patients underwent TACE (11 cases of hepatic carcinoma, seven cases of pancreatic carcinoma, and four cases of metastatic lymphadenopathy), 14 patients received radiotherapy, and 13 patients accepted brachytherapy after PTCD. The survival rate of the local tumor treatment group at 1, 3, 6, and 12 months was 97.96%, 95.92%, 89.80%, and 32.59% respectively; this was significantly longer than the survival times seen in the group receiving no adjuvant therapy [11]. The patency rates of stents and plastic catheters in the local tumor treatment group at 1, 3, 6, and 12 months were 97.96%, 93.86%, 80.93%, and 56.52% respectively; this again represents an improvement on rates in the non-therapy group [11].

TACE is the most widely used palliative treatment for unresectable HCC [22], while superselective intra-arterial chemotherapy with gemcitabine and embolization of tumor-feeding arteries have achieved positive results in unresectable CCA [13, 23]. Superselective intra-arterial chemotherapy and embolization of tumor-feeding arteries is also recommended as an attractive strategy for the treatment of unresectable gallbladder cancer [14]. In present study, we performed these locoregional tumor treatments according to the tumor location, size and pathologic diagnosis to prevent tumor growth. We achieved median stent patency times of 234 days (range 187–544 days) which is longer than those reported by Pai et al. [8]; the 1, 3, 6, 12 and 18 month survival rates were also better than those described by Qian et al. [11].

The present study does have several limitations, however. First, there is currently no method for monitoring the depth of tissue necrosis during ablation. We did not know the interval time of intraductal RFA nor the tolerance times of individual patients’ bile ducts; rather, we simply followed the parameters offered by the recently published case series. Second, with the aim of controlling tumor growth, we implemented locoregional tumor treatments in all patients, irrespective of the uncertain likelihood of local chemotherapies having effect on some tumors, such as extrahepatic CCA and gallbladder adenocarcinoma. The value of the above-mentioned local chemotherapy in treating such tumors warrants further investigation. Third, to our knowledge, this is the first report addressing tumor control after intraductal RFA in malignant biliary obstruction due to different tumors. The procedures studied here are complicated to perform and the optimal interval between intraductal RFA and locoregional tumor treatments is unclear; these factors may limit the use of this combined method as a treatment of choice for biliary stent re-occlusion and malignant biliary obstruction. Fourth, the number of patients in the study is limited because the intraductal RFA is a newly emerging technique and the implementation of intraductal RFA and locoregional tumor treatments have been limited in China due to economic concerns. With the same reason, the clinical relevance will be obvious weakening to analyze in CCA, HCC and gallbladder adenocarcinoma, separately. Prospective, randomized, single disease, and controlled studies with a large number of patients are underway to determine the efficacy of intraductal RFA followed by locoregional tumor treatments on long-term biliary stent patency and survival.

## Conclusions

In this group of patients with malignant biliary obstruction, intraductal RFA followed by locoregional tumor treatments for clearing occluded metal stents is safe and easy to perform, and can reestablish the canal with potential improved the stent-patency times and survival. Further large scale studies are needed to investigate in more detail the potential benefit offered by combination treatment.

## Supporting Information

S1 Fig Detail of PTC biopsy. (PDF)

S2 Fig The detail images of the RFA catheter. (PDF)
