# Supplementary material for: Intraductal Radiofrequency Ablation Followed by Locoregional Tumor Treatments for Treating Occluded Biliary Stents in Non-Resectable Malignant Biliary Obstruction: A Single-Institution Experience
Source: PLoS One. 2015 Aug 5;10(8):e0134857. doi: 10.1371/journal.pone.0134857 (PMC4526692; doi:10.1371/journal.pone.0134857)

PTC biopsy

All patients underwent PTC and percutaneous transhepatic cholangiography biopsy (PTCB) before stent implantation in the initial procedure. The procedure was performed by transhepatic puncture of a branch of the intrahepatic bile duct with a 21-gauge needle, under digital subtraction angiography (DSA) guidance (Artis Zeego, Siemens, Munich, Germany). PTC was then performed to visualize the location, extent and degree of bile duct obstruction using a percutaneous transhepatic cholangiography puncture set (Cook Inc., Bloomington, IN, USA). A 5 F Cobra catheter (Cook Inc.) was introduced into the bile duct with a 0.035 inch guide wire (Cook Inc.). The catheter and guide wire were passed through the stenosis simultaneously into the duodenum or jejunum. The guide wire was then replaced with a 0.035 inch stiff guide wire (Cook Inc.), and a 9 F catheter sheath (Figure 1a, b) was advanced along the stiff guide wire with 8 F biopsy forceps (Figure 2a, b) passed through the sheath. The sheath was pushed forward forcefully against the upper segment of the stenosis, while the biopsy forceps were introduced to the lesion through the introducing sheath. PTCB was undertaken using fluoroscopic guidance. The biopsy forceps were pushed forward forcefully against the upper segment of the stenosis, while the biopsy forceps grasped the lesion through the introducing sheath (Figure 3a, b). The biospsy forceps was opened and pushed forward 5-10mm, then closed and retracted to tear out a tissue specimen (Figure 4a, b, c).

Additional figures

Figure 1a, b. The imaging of a 9F long sheath.

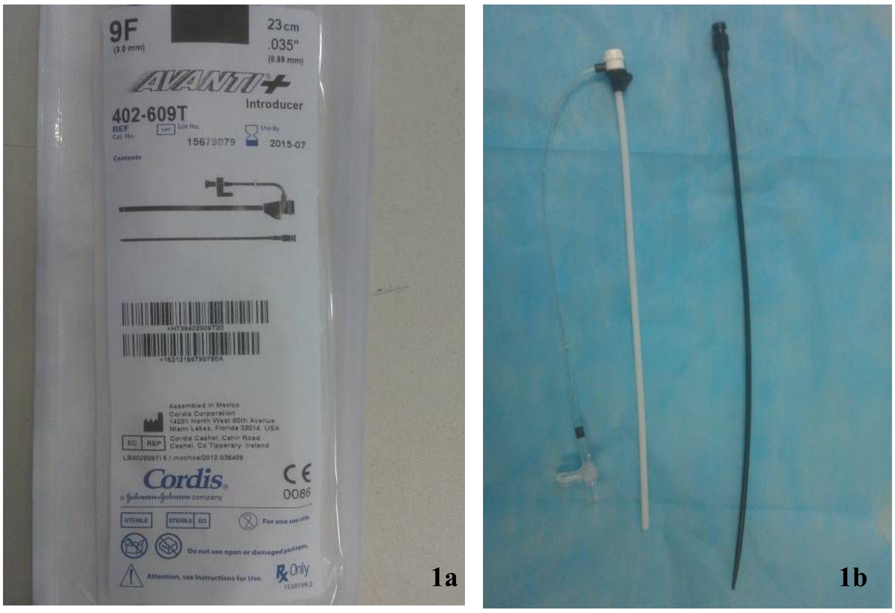

Figure 2a/2b. The biopsy forceps for biliary tract.

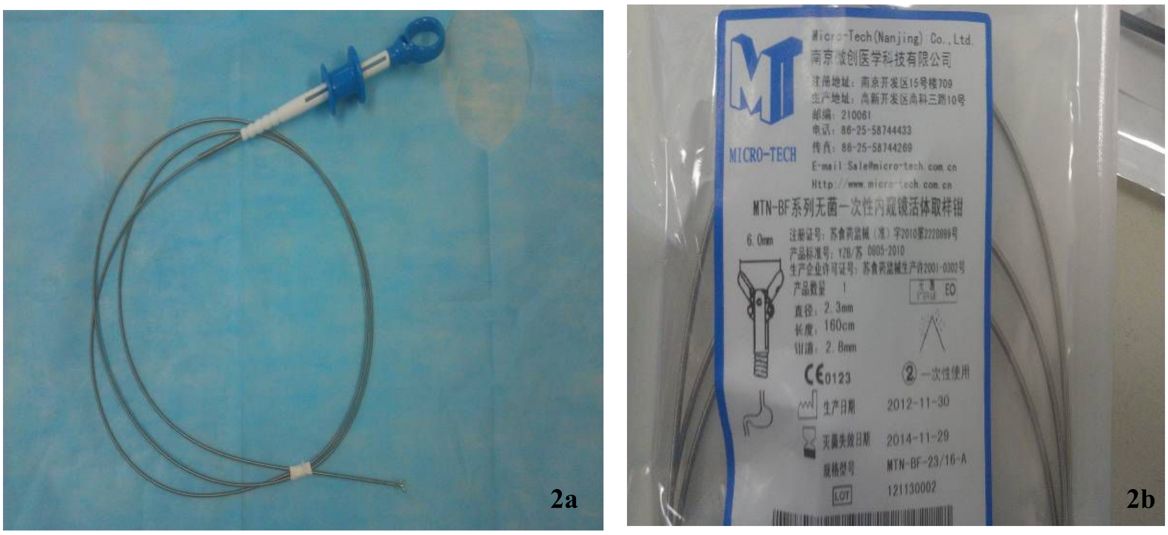

Figure 3a, b. The image of the position relation about the biopsy forceps, 0.035 inch guide wire and 9F sheath.

3a

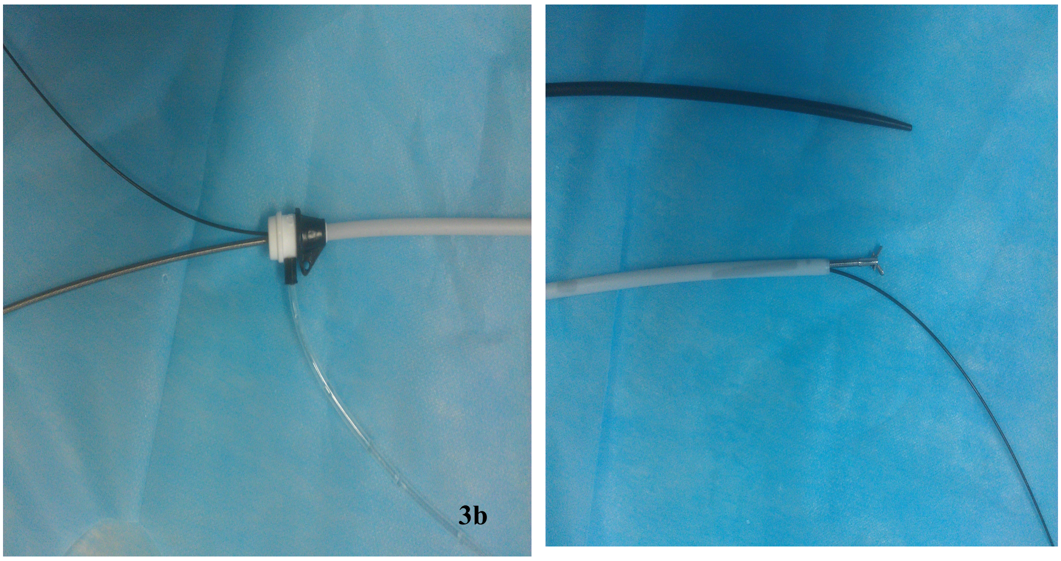

Figure 4a, b, c. The operation procedureof biopsy.

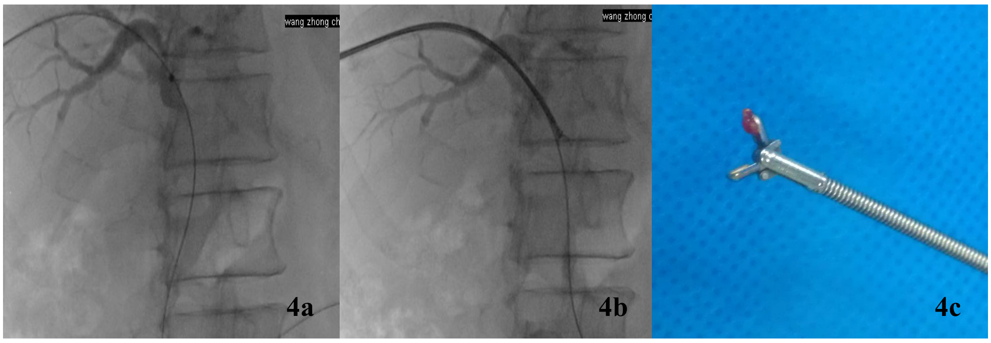

Supplement: S1 Fig — (PDF) [file pone.0134857.s001.pdf]
