# Supplementary material for: Intraductal Radiofrequency Ablation Followed by Locoregional Tumor Treatments for Treating Occluded Biliary Stents in Non-Resectable Malignant Biliary Obstruction: A Single-Institution Experience
Source: PLoS One. 2015 Aug 5;10(8):e0134857. doi: 10.1371/journal.pone.0134857 (PMC4526692; doi:10.1371/journal.pone.0134857)

Figure 1 is the sketch map of intraductal RFA.

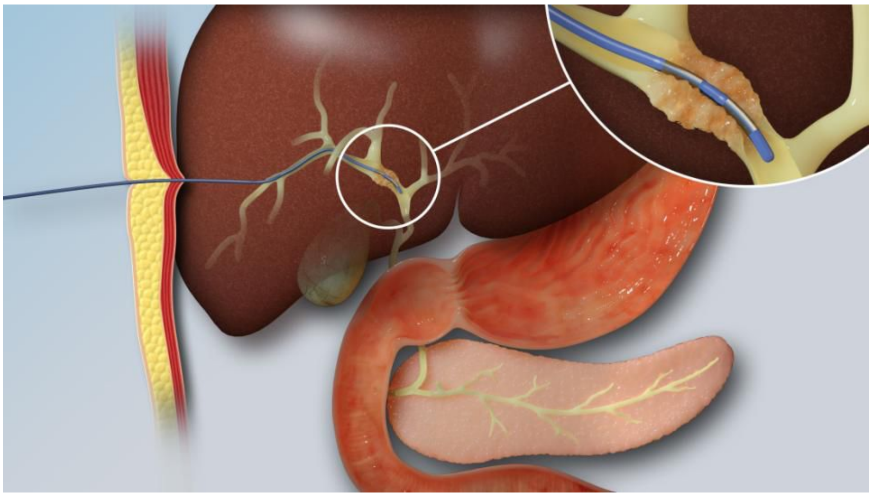

Figure 2 Close up of the Habib TM Percutaneous Endobiliary Radiofre-quency (HabibTM PERF) catheters showing the two spirals cut electrodes, 8-mm spacing, with the distal electrode 5 mm from the tip.

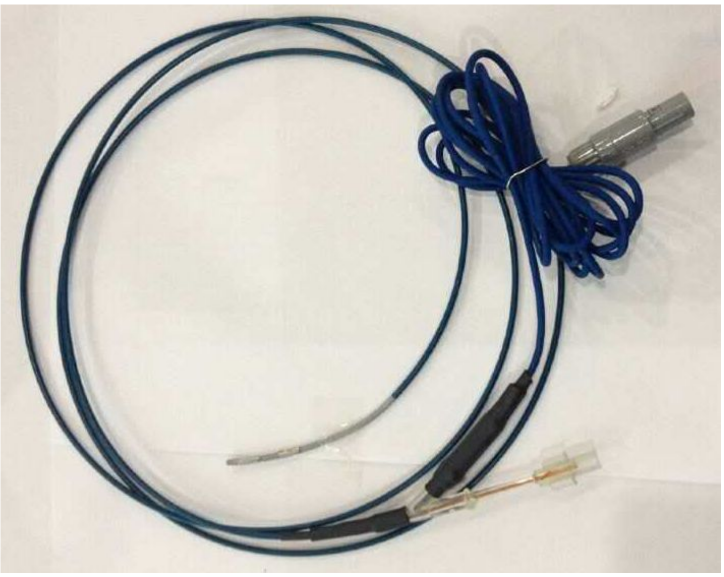

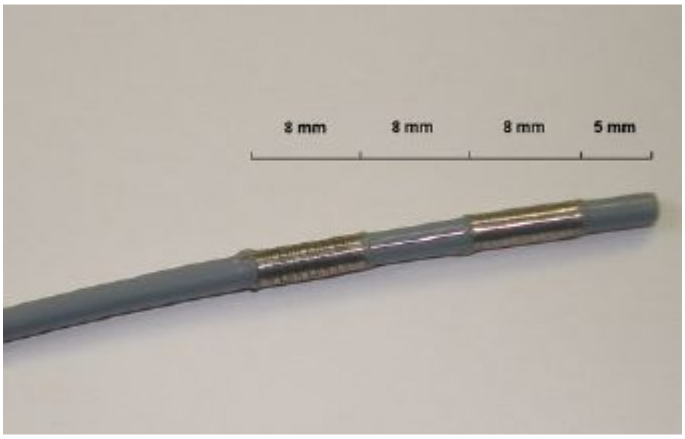

Supplement: S2 Fig — (PDF) [file pone.0134857.s002.pdf]
